# Mapping the General Fertility Rate in Greece: A Time-Trend Nationwide Analysis (1956-2024)

**DOI:** 10.7759/cureus.114258

**Published:** 2026-08-10

**Authors:** Chrysi Christodoulaki, Nikolaos Vlachadis, Charalampos Theofanakis, Rafaela Panagopoulou, Ioanna Petrakou, Dimos Sioutis, Nikolaos Machairiotis, Periklis Panagopoulos

**Affiliations:** 1 Department of Obstetrics and Gynecology, General Hospital of Chania, Chania, GRC; 2 Third Department of Obstetrics and Gynecology, National and Kapodistrian University of Athens, Medical School, Attiko University Hospital, Athens, GRC; 3 Postgraduate Program "High-Risk Pregnancy", National and Kapodistrian University of Athens, Medical School, Attiko University Hospital, Athens, GRC

**Keywords:** births, fertility, fertility rates, general fertility rate, greece, time trends, women of childbearing age, women of reproductive age

## Abstract

Background: Accurate assessment of fertility rates and their long-term trends is essential for understanding reproductive dynamics, anticipating the healthcare and socioeconomic consequences of demographic change, and informing family planning and population policies. The aim of this study was to investigate long-term trends in the general fertility rate (GFR) in Greece using nationwide population data.

Materials and methods: Official national data on annual live births and the female population aged 15-44 years were obtained from the Hellenic Statistical Authority for the period 1956-2024. The GFR was calculated as the annual number of live births per 1,000 women of reproductive age (WRA). An age-standardized GFR (ASGFR) was also calculated for each study year using the 2013 European Standard Population. Temporal trends were analyzed using Joinpoint regression to estimate annual percent changes (APCs) and corresponding 95% confidence intervals (95% CIs).

Results: The GFR declined during 1956-1980 (APC = -0.3, 95% CI: -0.4 to -0.2), with two historic peaks recorded in 1959 and 1967, when it reached 81.6 and 80.7 births per 1,000 women aged 15-44 years, respectively. Over the subsequent two decades, the GFR declined further (1980-1989: APC = -4.7, 95% CI: -5.5 to -4.2; 1989-1999: APC = -1.2, 95% CI: -1.6 to -0.7). From 1999 to 2009, the GFR increased (APC = 2.1, 95% CI: 1.7 to 2.6). Thereafter, it entered a renewed period of decline, reaching a historic low of 39.4 births per 1,000 women aged 15-44 years in 2024, representing a 25% decrease compared with 2009. This decline was characterized by two periods of significant reduction, during 2009-2013 (APC = -3.7, 95% CI: -5.4 to -2.0) and 2021-2024 (APC = -5.0, 95% CI: -8.1 to -3.0), with a period of relative stability between 2013 and 2021. Following a period of stability during 1956-1963, the ASGFR increased during 1963-1967 (APC = 2.6, 95% CI: 1.3 to 4.1) and subsequently declined during 1967-1999. The rate increased again during 1999-2009 (APC = 2.3, 95% CI: 1.9 to 2.7), declined during 2009-2013 (APC = -3.4, 95% CI: -5.9 to -0.7) and 2021-2024 (APC = -4.4, 95% CI: -6.9 to -1.8), whereas it showed a modest upward trend during 2013-2021 (APC = 0.9, 95% CI: 0.2 to 1.7). During 2009-2024, approximately 70% of the overall decline in the GFR was attributable to declining fertility rates and 30% to changes in the age composition of women of reproductive age. However, the contribution of these factors shifted over time: alterations in the age distribution of women of reproductive age accounted for most of the decline during 2009-2021, whereas fertility decline became the predominant driver during 2021-2024.

Conclusions: This nationwide study demonstrates a sustained long-term decrease in the GFR in Greece over nearly seven decades, culminating in a historic low in 2024, despite a temporary recovery between 1999 and 2009. The recent GFR downward trend observed from 2009 to 2024 was the result of fertility decline, exacerbated by unfavorable shifts in the age structure of women of reproductive age.

## Introduction

Over the past several decades, fertility has declined substantially worldwide, while life expectancy has continued to increase, resulting in major demographic changes. Since 1950, global fertility has fallen by more than 50%, with the decline progressively spreading from high-income to middle- and low-income countries [1-3]. During the past two decades, fertility rates have reached historically low levels in most countries, accompanied by a continuing increase in the age at childbearing [1]. The transition to low fertility has been largely attributed to socioeconomic development and urbanization, which have profoundly changed women's roles in society. Contributing factors include higher educational attainment, increased labor force participation among women, and widespread access to effective contraception. Traditionally, fertility has followed economic cycles, declining during periods of recession and recovering during periods of economic expansion. In recent decades, however, fertility has declined more sharply during economic downturns and recovered only modestly during subsequent periods of economic growth [2-5]. Accumulating evidence indicates that persistent very low fertility reflects the combined effects of structural socioeconomic changes, unstable labor markets, recurrent crises, uncertainty about the future, changing partnership patterns, and evolving attitudes toward parenthood and reproductive preferences. Compared with previous generations, young adults report lower and more flexible fertility intentions, and their reproductive decisions are increasingly shaped by uncertainty about future living conditions. Together, these factors have contributed to delayed childbearing and declining family size [2,6,7]. Postponement of parenthood also has important biological implications, as increasing maternal and paternal age is associated with a higher risk of infertility. Moreover, biological and environmental factors, including obesity, lifestyle-related factors, and exposure to reproductive toxicants, have adversely affected natural fertility despite substantial advances in assisted reproductive technology (ART) [4,5,8,9].

Accurate assessment of fertility rates and their long-term trends is essential for evaluating a country's reproductive dynamics and anticipating the healthcare, social, and economic challenges associated with demographic change. Reliable estimates and projections of fertility indicators are also necessary to inform healthcare planning, family planning, and population policies [1,8,10]. Greece is among the European countries experiencing a sustained decline in the number of births, making the continuous monitoring of fertility indicators particularly important. A recent study investigated temporal trends in births in Greece over seven decades since 1950 [11]. Although trends in the absolute number of live births provide valuable information on demographic change, they do not account for temporal changes in the size of the population of women of reproductive age (WRA). In contrast, the general fertility rate (GFR) relates the annual number of live births to the population of women of reproductive age and therefore provides a more informative assessment of fertility trends. The aim of the present study was to investigate long-term trends in the GFR in Greece using nationwide population data.

## Materials and methods

Study population and parameters

Official national data on live births in Greece by 5-year maternal age groups (15-19, 20-24, 25-29, 30-34, 35-39, and 40-44 years), together with corresponding mid-year population estimates of women in these age groups for the period 1956-2024, were obtained from the Hellenic Statistical Authority [12]. Complete annual nationwide data were available throughout the entire study period. The registration of live births by maternal age is highly complete (>99.9%), as previously documented [13]. For each year between 1956 and 2024, the GFR was calculated as the number of live births to women aged 15-44 years divided by the estimated mid-year population of women of reproductive age (WRA; 15-44 years) [14]. GFR values were expressed as live births per 1,000 WRA. To account for temporal changes in the age composition of the WRA population, an age-standardized general fertility rate (ASGFR) was calculated for each year using the direct method of standardization, with the 2013 European Standard Population as the reference population. This contemporary European reference facilitates the comparison of age-standardized estimates over time and across European populations [15]. The WRA population was defined as women aged 15-44 years because this age range better reflects natural fertility and is consistent with the definition used by the Centers for Disease Control and Prevention (CDC) [16].

Statistical analysis

Data management and calculation of fertility indicators were performed using Microsoft Excel 2010 (Microsoft Corporation, Redmond, WA). Temporal trends were evaluated using the Joinpoint Regression Program version 6.0.0 (National Cancer Institute, Bethesda, MD). This method identifies statistically significant changes in temporal trends by fitting segmented log-linear regression models. The maximum number of joinpoints permitted in each model was set at six. Model selection was performed using Monte Carlo permutation tests. Analyses were based on the assumptions of homoscedastic errors and absence of autocorrelation. For each trend segment identified by the model, annual percent changes (APCs) and their corresponding 95% confidence intervals (95% CIs) were estimated. To summarize trends over longer time intervals comprising multiple segments, average annual percent changes (AAPCs) were also calculated. Statistical significance was assessed using two-sided t-tests, with p-values < 0.05 considered statistically significant.

To facilitate a more detailed evaluation of temporal trends over the extended study period (1956-2024), trend analyses were conducted separately for two periods. The year 1999 was selected as the dividing point because it represented a local minimum in the GFR and marked the transition from a prolonged period of fertility decline to a subsequent period characterized by different fertility dynamics. Accordingly, the study period was divided into 1956-1999 and 1999-2024.

The contribution of changes in the age composition of the WRA population to temporal trends in the GFR was assessed by comparing percentage changes in the crude GFR with the corresponding percentage changes in the ASGFR. Because the ASGFR is standardized to the 2013 European Standard Population, changes in this measure reflect changes in age-specific fertility independent of shifts in the age distribution of women of reproductive age. Accordingly, the difference between the percentage changes in the GFR and ASGFR was interpreted as the contribution of changes in the age composition of the WRA population. These findings were corroborated using the decomposition method described by Siegel and Swanson, which accounts for the interaction component between changes in age-specific fertility rates and the age composition of women of reproductive age [17].

## Results

**Figure 1:**
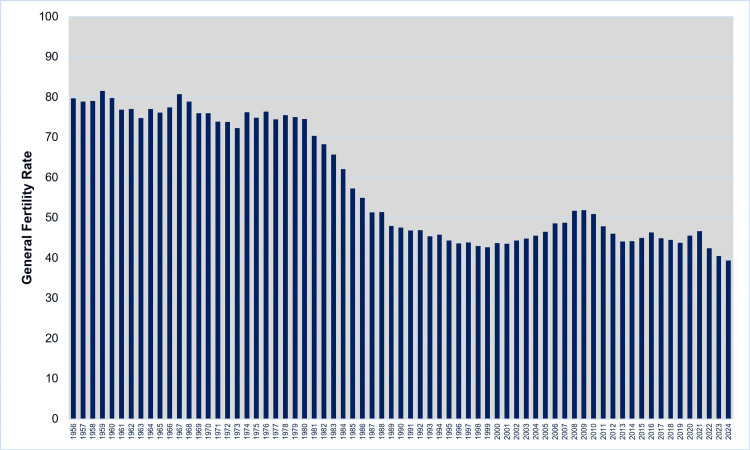
General fertility rate (per 1,000 women aged 15-44 years) in Greece, 1956-2024

The GFR exhibited two historic peaks in 1959 and 1967, reaching 81.6 and 80.7 births per 1,000 WRA, respectively, corresponding to 159,002 and 162,373 live births among women aged 15-44 years. Over the subsequent three decades, the GFR declined to 42.7 births per 1,000 WRA in 1999, the lowest value recorded during the 20th century. During the following decade, the GFR increased to 51.9 births per 1,000 WRA in 2009, before resuming its downward trajectory and ultimately reaching a historic low of 39.4 births per 1,000 WRA in 2024 (Figure 1).

**Figure 2:**
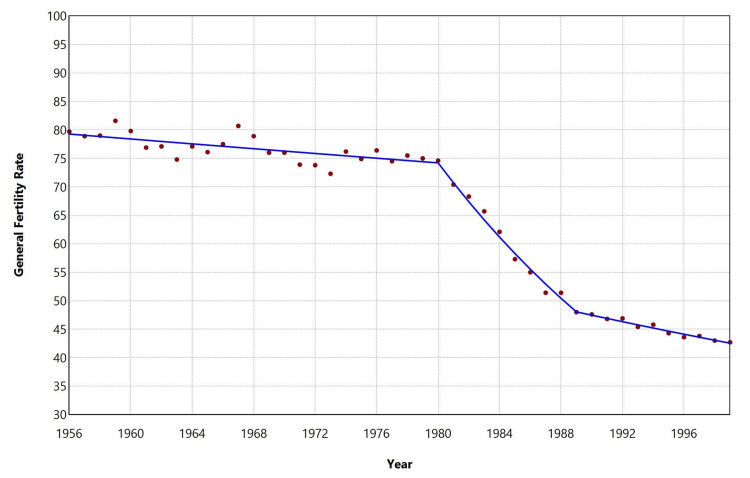
Trends in the general fertility rate (per 1,000 women aged 15-44 years) in Greece, 1956-1999

Joinpoint regression analysis showed that the GFR declined gradually during 1956-1980 (APC = -0.3, 95% CI: -0.4 to -0.2). This was followed by two distinct phases of decline: a period of rapid decrease during 1980-1989 (APC = -4.7, 95% CI: -5.5 to -4.2) and a slower decline during 1989-1999 (APC = -1.2, 95% CI: -1.6 to -0.7). Overall, the GFR decreased during 1956-1999, with an AAPC of -1.4% (95% CI: -1.5% to -1.4%). Notably, the reduction observed during 1980-1989 alone accounted for approximately 70% of the overall decline in the GFR between 1967 and 1999 (Figure 2, Table 1).

**Table 1:** Trends in the general fertility rate in Greece, 1956-2024 Trends and the corresponding p-values were calculated using joinpoint regression analysis. *Statistically significant

Segment	Annual percent change	95% confidence interval	p-value
1956-1980	-0.3	-0.4 to -0.2	<0.001*
1980-1989	-4.7	-5.5 to -4.2	<0.001*
1989-1999	-1.2	-1.6 to -0.7	<0.001*
1999-2009	2.1	1.7 to 2.6	0.001*
2009-2013	-3.7	-5.4 to -2.0	0.002*
2013-2021	0.3	-0.3 to 2.5	0.297
2021-2024	-5.0	-8.1 to -3.0	<0.001*

**Figure 3:**
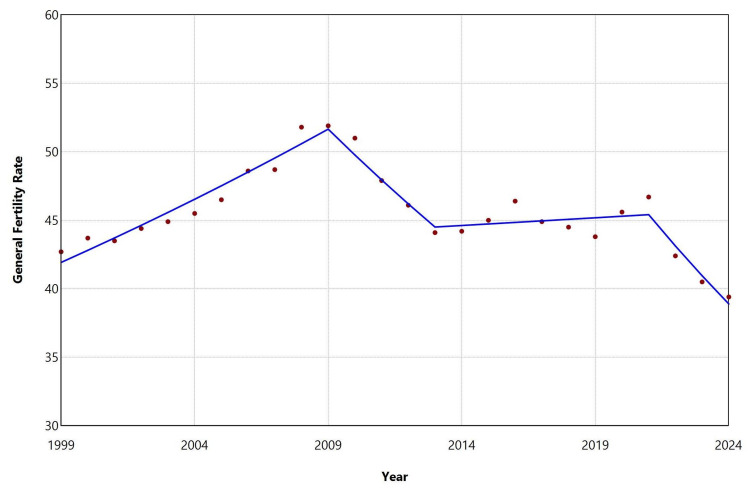
Trends in the general fertility rate (per 1,000 women aged 15-44 years) in Greece, 1999-2024

From 1999 to 2009, Greece experienced the only sustained increase in the GFR observed during the study period, with an APC of 2.1 (95% CI: 1.7 to 2.6). Thereafter, the GFR resumed its downward trend, characterized by two periods of significant decline: 2009-2013 (APC = -3.7, 95% CI: -5.4 to -2.0) and 2021-2024 (APC = -5.0, 95% CI: -8.1 to -3.0), separated by a period of relative stability between 2013 and 2021. Overall, the GFR decreased during 2009-2024 with an AAPC of -1.9% (95% CI: -2.2% to -1.5%). Approximately 60% of the overall reduction during this period occurred in the final three years of the study (2021-2024) (Figure 3, Table 1).

**Figure 4:**
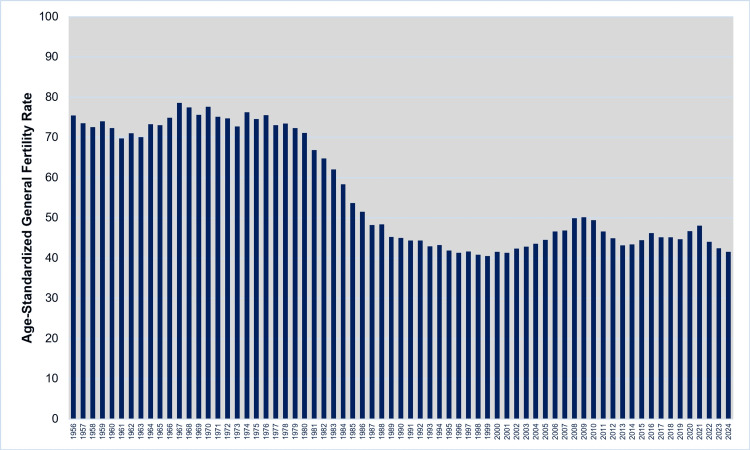
Age-standardized general fertility rate (per 1,000 women aged 15-44 years) in Greece, 1956-2024

The ASGFR reached its highest value in 1967, at 78.6 births per 1,000 WRA. Thereafter, the rate declined to its historic minimum of 40.5 births per 1,000 WRA in 1999 before increasing to 50.2 births per 1,000 WRA in 2009. Following this temporary recovery, the ASGFR resumed its downward trajectory, reaching 41.5 births per 1,000 WRA in 2024 (Figure 4).

**Figure 5:**
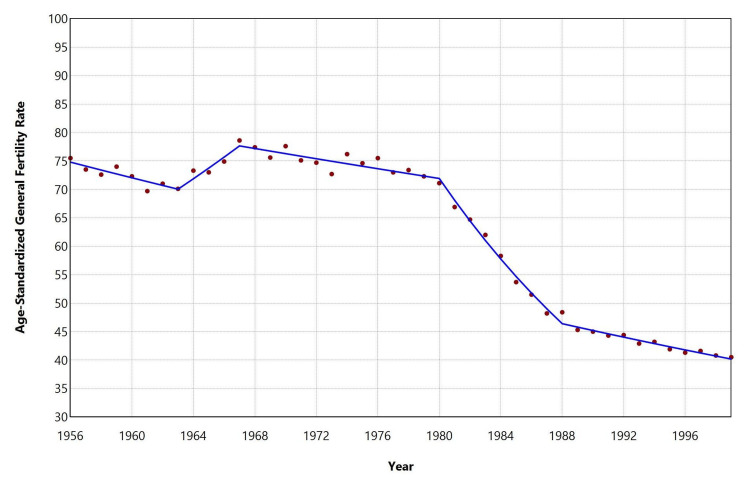
Trends in the age-standardized general fertility rate (per 1,000 women aged 15-44 years) in Greece, 1956-1999

Joinpoint regression analysis showed that the ASGFR generally declined between 1960 and 1999, with the exception of the 1963-1967 period, during which it increased (APC = 2.6, 95% CI: 1.3 to 4.1). The steepest decline was observed during 1980-1988 (APC = -5.3, 95% CI: -6.0 to -4.8). This was followed by a period of recovery between 1999 and 2009, when the ASGFR increased at an APC of 2.3 (95% CI: 1.9 to 2.7). Overall, the ASGFR decreased during 1956-1999, with an AAPC of -1.4% (95% CI: -1.5% to -1.4%) (Figure 5, Table 2).

**Table 2:** Trends in the age-standardized general fertility rate in Greece, 1956-2024 Trends and the corresponding p-values were calculated using joinpoint regression analysis *Statistically significant

Segment	Annual percent change	95% confidence interval	p-value
1956-1963	-0.9	-1.9 to -0.4	0.002*
1963-1967	2.6	1.3 to 4.1	<0.001*
1967-1980	-0.6	-0.9 to -0.4	<0.001*
1980-1988	-5.3	-6.0 to -4.8	<0.001*
1988-1999	-1.3	-1.6 to -0.9	<0.001*
1999-2009	2.3	1.9 to 2.7	<0.001*
2009-2013	-3.4	-5.9 to -0.7	0.016*
2013-2021	0.9	0.2 to 1.7	0.014*
2021-2024	-4.4	-6.9 to -1.8	0.003*

**Figure 6:**
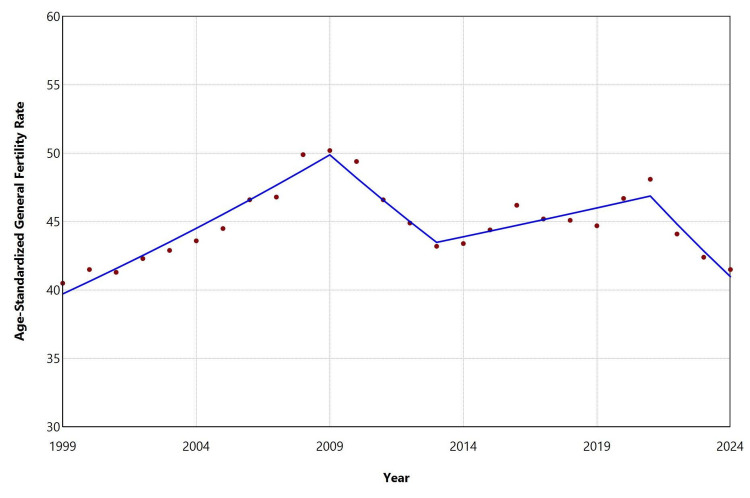
Trends in the age-standardized general fertility rate (per 1,000 women aged 15-44 years) in Greece, 1999-2024

During 2009-2024, the ASGFR resumed its downward trend. This decline was characterized by two periods of significant reduction (2009-2013: APC = -3.4, 95% CI: -5.9 to -0.7; 2021-2024: APC = -4.4, 95% CI: -6.9 to -1.8), separated by a modest increase during 2013-2021 (APC = 0.9, 95% CI: 0.2 to 1.7). Overall, the ASGFR decreased during 2009-2024, with an AAPC of -1.4% (95% CI: -1.7% to -1.1%) (Figure 6, Table 2).

Between 1967 and 1999, the GFR decreased by 47%, from 80.7 to 42.7 births per 1,000 WRA, a decline that closely mirrored the 48% reduction in the ASGFR, from 78.6 to 40.5 births per 1,000 WRA. These findings indicate that the decline in the GFR during this period was driven almost entirely by decreasing age-standardized fertility rates, while changes in the age composition of the WRA population slightly attenuated the decline in the GFR. During 1999-2009, the GFR increased by 22%, from 42.7 to 51.9 births per 1,000 WRA, a trend that was similarly reflected in the ASGFR, which rose by 24% from 40.5 to 50.2 births per 1,000 WRA. The slightly smaller increase in the GFR is consistent with the gradual aging of the WRA population during this period.

From 2009 to 2024, the GFR declined by 25%, substantially more than the 17% reduction observed in the ASGFR, which decreased from 50.2 to 41.5 births per 1,000 WRA. Thus, approximately 70% of the decline in the GFR was attributable to declining fertility rates, whereas the remaining 30% reflected aging of the WRA population. However, the relative contributions of declining fertility rates and WRA population aging to the observed decline in the GFR changed markedly over the period 2009-2024. During 2009-2021, the GFR declined by 10% (from 51.9 to 46.7 births per 1,000 WRA), whereas the ASGFR declined by only 4% (from 50.2 to 48.1 births per 1,000 WRA). Accordingly, approximately 60% of the observed decline in the GFR during this period was attributable to aging of the WRA population, whereas only around 40% resulted from declining fertility rates. This pattern changed markedly during 2021-2024. Over this period, the GFR declined 16%, from 46.7 to 39.4 births per 1,000 WRA, while the ASGFR decreased 14%, from 48.1 to 41.5 births per 1,000 WRA. Accordingly, the vast majority (~90%) of the decline in the GFR during 2021-2024 was attributable to declining fertility rates, whereas only approximately 10% was explained by continued aging of the WRA population (Table 3).

**Table 3:** Contributors to the observed decline in the general fertility rate in Greece, by period, 2009-2021 GFR: general fertility rate, ASGFR: age-standardized general fertility rate, WRA: women of reproductive age (15-44 years)

Segment	GFR decrease	ASGFR share	WRA age structure share
2009-2021	10%	41%	59%
2021-2024	15%	87%	13%
2009-2024	25%	71%	29%

## Discussion

The present study provides a comprehensive assessment of long-term fertility trends in Greece over nearly seven decades, incorporating both crude and age-standardized measures of the GFR. Several important findings emerged. Fertility declined markedly between the late 1960s and the end of the 20th century, with the GFR decreasing by approximately one-half between 1967 and 1999. This was followed by a temporary recovery during 1999-2009, representing the only sustained period of increasing fertility observed throughout the study period. After 2009, however, the GFR declined once again, reaching historically low levels by 2024. Comparison of trends in the crude and age-standardized GFR also highlighted the important contribution of changes in the age composition of the WRA population to the decline in the GFR observed after 2009.

The decline in fertility between 1967 and 1999 was substantial, with the 1980s representing the period of the most rapid reduction. Although the GFR partially recovered during 1999-2009, this improvement proved temporary and was completely reversed following the onset of the economic crisis. From 2022 onward, Greece experienced successive record-low fertility levels, indicating a recent acceleration in the long-term downward trend. These findings suggest that the modest recovery observed during the early 2000s was insufficient to offset the sustained decline in fertility and that the deterioration in recent years has been particularly pronounced.

The long-term decline in fertility observed in Greece is consistent with broader demographic developments across Europe and other high- and middle-income countries. In the United States, the GFR decreased 45% from 1960 to 2002 [18]. Fertility has decreased substantially throughout much of the developed world since the second half of the 20th century, reflecting profound social, economic, and cultural transformations [1,2]. The temporary increase in fertility observed between 1999 and 2009 interrupted this long-term decline. Previous studies have suggested that this rebound was driven, at least in part, by the postponement transition, as women who had delayed childbearing during the 1990s subsequently realized their fertility intentions during a period of relatively favorable economic conditions [19]. However, this temporary improvement was completely reversed following the onset of the economic crisis in 2009, after which the GFR resumed its downward trajectory and continued to decline through 2024.

To further characterize long-term fertility trends independently of changes in the age composition of the WRA population, the ASGFR was calculated for each study year. This analysis showed that age-standardized fertility exhibited statistically significant increasing trends not only during 1999-2009 but also during 1963-1967 and 2013-2021. Interestingly, each of these three distinct periods of increasing fertility was followed by an abrupt reversal that coincided with major national events: the establishment of the military dictatorship in 1967, the onset of the economic crisis in 2009, and the COVID-19 pandemic in 2021 [11]. These temporal associations suggest that major societal disruptions may have interrupted periods of improving fertility in Greece. Notably, the ASGFR reached its highest value in 1967, the same year in which the country recorded the historically highest number of live births [11], whereas the lowest value of the GFR was observed in 1999.

The age-standardized analysis of the GFR also provided insight into the contribution of changes in the age composition of the WRA population to long-term fertility trends. The findings showed that both the decline observed during 1967-1999 and the subsequent increase during 1999-2009 were almost entirely driven by changes in age-specific fertility rates. In fact, changes in the age composition of the WRA population slightly attenuated both trends. In contrast, after 2009, the shift in the age distribution of the WRA population toward older, less fertile age groups acted synergistically with declining fertility rates, reinforcing the decline in the GFR. Across the entire 2009-2024 period, changes in the age composition of the WRA population accounted for almost one-third of the decline in the GFR. Their contribution was particularly pronounced during 2009-2021, whereas the sharp decline observed between 2021 and 2024 was predominantly behavioral, reflecting substantial reductions in fertility rates.

This study has several important strengths. It was based on complete national data spanning nearly seven decades, providing comprehensive population coverage, minimizing the risk of selection bias, and enhancing the representativeness and reliability of the findings. In contrast to studies that examine trends in the absolute number of live births, the present study analyzed trends in the GFR, a measure that accounts for the size of the population of women of reproductive age and therefore provides a more informative assessment of fertility trends [17]. Although the GFR accounts for changes in the size of the WRA population, it remains influenced by its age composition, as fertility rates vary substantially across reproductive ages. Therefore, the GFR was age-standardized using the direct method with the European Standard Population as the reference population, allowing long-term fertility trends to be assessed independently of changes in the age composition of the WRA population. This approach ensured longitudinal consistency and enhanced the international comparability of the findings. Finally, the combined analysis of crude and age-standardized GFRs enabled the distinction between changes in underlying fertility and changes attributable to shifts in the age composition of the population of women of reproductive age, thereby providing novel insights into the drivers of long-term fertility trends in Greece.

Several limitations of the present study should be acknowledged. First, the analysis was based on aggregated national data and therefore did not allow the assessment of regional variations or individual-level determinants of fertility, including parity, migration status, educational level, marital status, use of ART, and other socioeconomic characteristics. Second, the WRA population, which constituted the denominator of the GFR, was defined as women aged 15-44 years [16]. Although some studies calculate the GFR using women aged 15-49 years [17], the present study focused on the age range representing natural fertility. Births to women aged 45-49 years remain relatively uncommon, despite their gradual increase in recent years, and including this age group would have diluted the GFR without materially affecting the interpretation of long-term fertility trends. Moreover, the vast majority of births among women aged 45-49 years result from the use of ART [20]. Third, the mid-year estimates of the WRA population were derived from official population estimates based on population census data and estimated population inflows and outflows due to migration and are therefore subject to some degree of estimation error. Furthermore, possible methodological revisions over this extended period may have introduced additional uncertainty into the population estimates. Nevertheless, a sensitivity analysis using a formal decomposition approach produced comparable estimates and did not materially alter the conclusions or their interpretation [17]. As this analysis was performed solely to assess the robustness of the findings, the detailed results are not presented because they fall beyond the primary scope of the present study.

Focusing on the most recent period, Greece experienced a dramatic decline in the GFR after 2009, which accelerated during the final three years of the study period (2021-2024). This recent deterioration coincided with the adverse international economic environment that followed the COVID-19 pandemic [21], culminating in the lowest GFR ever recorded in Greece in 2024. The decline in the GFR reflects the combined effects of declining fertility rates and an unfavorable shift in the age composition of the WRA population. Importantly, this shift should not be interpreted simply as an increase in the proportion of women at older reproductive ages. Rather, it reflects a redistribution of the WRA population away from the peak reproductive ages (25-34 years), where fertility is highest, toward age groups with lower fertility. These findings suggest that Greece may have entered a new phase of its fertility transition, characterized not only by an increasingly unfavorable demographic structure but also by a renewed deterioration in fertility behavior. Although the factors underlying this recent decline require further investigation, persistent economic uncertainty, housing affordability challenges, delayed family formation, and broader societal changes influencing reproductive decision-making may represent potential contributing factors.

## Conclusions

This nationwide study demonstrated a sustained long-term decline in the GFR in Greece over nearly seven decades, despite a temporary recovery during 1999-2009, with the post-2009 decline accelerating markedly and reaching a historic low in 2024. The findings indicate that this decline resulted from the combined effects of declining age-specific fertility rates and an unfavorable shift in the age composition of the WRA population, suggesting that Greece has entered a new phase of its fertility transition characterized by both structural and behavioral changes. Further research is needed to investigate the socioeconomic and demographic determinants of the recent fertility decline and barriers to achieving reproductive intentions, while policies promoting family formation should address key areas such as family support, reproductive health, and fertility awareness.
